# Multipath-Assisted Radio Sensing and State Detection for the Connected Aircraft Cabin †

**DOI:** 10.3390/s22082859

**Published:** 2022-04-08

**Authors:** Jonas Ninnemann, Paul Schwarzbach, Michael Schultz, Oliver Michler

**Affiliations:** 1 Institute of Traffic Telematics, Technische Universität Dresden, 01069 Dresden, Germany; paul.schwarzbach@tu-dresden.de (P.S.); oliver.michler@tu-dresden.de (O.M.); 2 Institute of Flight Systems, Bundeswehr University Munich, 85577 Neubiberg, Germany; michael.schultz@unibw.de

**Keywords:** multipath-assisted radio sensing (MARS), wireless sensor network (WSN), channel impulse response (CIR), ultra-wideband (UWB), beyond 5G (B5G), connected aircraft cabin, aircraft boarding, occupancy detection, probabilistic grid mapping, k-nearest neighbor (kNN) classification

## Abstract

Efficiency and reliable turnaround time are core features of modern aircraft transportation and key to its future sustainability. Given the connected aircraft cabin, the deployment of digitized and interconnected sensors, devices and passengers provides comprehensive state detection within the cabin. More specifically, passenger localization and occupancy detection can be monitored using location-aware communication systems, also known as wireless sensor networks. These multi-purpose communication systems serve a variety of capabilities, ranging from passenger convenience communication services, over crew member devices, to maintenance planning. In addition, radio-based sensing enables an efficient sensory basis for state monitoring; e.g., passive seat occupancy detection. Within the scope of the connected aircraft cabin, this article presents a multipath-assisted radio sensing (MARS) approach using the propagation information of transmitted signals, which are provided by the channel impulse response (CIR) of the wireless communication channel. By performing a geometrical mapping of the CIR, reflection sources are revealed, and the occupancy state can be derived. For this task, both probabilistic filtering and k-nearest neighbor classification are discussed. In order to evaluate the proposed methods, passenger occupancy detection and state detection for the future automation of passenger safety announcements and checks are addressed. Therefore, experimental measurements are performed using commercially available wideband communication devices, both in close to ideal conditions in an RF anechoic chamber and a cabin seat mockup. In both environments, a reliable radio sensing state detection was achieved. In conclusion, this paper provides a basis for the future integration of energy and spectrally efficient joint communication and sensing radio systems within the connected aircraft cabin.

## 1. Introduction

The performance of airline operations is driven by appropriate air traffic management and efficient ground handling. The seamless coupling of the vehicle (aircraft) and passenger trajectories in particular represents one of the greatest challenges in future mobility management. As is currently very evident, pandemic passenger transport requirements are having a significant impact on the performance of the entire transportation network. The effective and efficient management of passenger handling processes at bottlenecks is the key to operational improvements. A crucial problem in air travel is efficient passenger handling (e.g., boarding, disembarkation) in the confined aircraft cabin.

Digitalization provides fundamental tools for state detection and monitoring within the cabin. In addition, interconnection and communication capabilities enable distributed state perception and centralized monitoring and controlling. A digital, fully connected aircraft cabin is the mandatory infrastructure needed to facilitate cabin operations and enable and significantly support effective passenger management [1,2]. These use cases range from improving the passenger experience, over the optimization of boarding and crew operations, to maintenance planing. This technology begins with a high data rate wireless communication network for connecting different devices in the cabin, such as passenger handheld devices, crew member controlling devices, in-flight entertainment systems or maintenance sensors.

For these tasks, wireless sensor networks (WSNs) provide a cheap, energy and infrastructure-efficient sensory basis. Next to the informational interconnection, WSNs are used for the active localization of objects and persons, especially in GNSS-denied environments. Within the context of the connected aircraft cabin [3,4], this could include passenger and crew member localization, as well as the galley trollies. Furthermore, the integrated use of WSNs also enables radio sensing functionalities for radar-like imaging and surveillance [5,6]. Radio sensing particularly offers a variety of application-related benefits, as target objects are not required to be equipped with dedicated hardware. Instead, the signal properties of the communication signal are analyzed to perform a radar-like detection of objects in the environment [7,8]. This approach enables an energy, spectral and cost-efficient solution for detection, passive localization and mapping [9]. Due to these advantages, radio sensing is discussed as one of the core features of beyond 5G mobile communication systems, especially 6G [10,11]. Within this context, the terms integrated sensing and communication [12], as well as joint communication and sensing, have been established [13].

**Figure 1:**
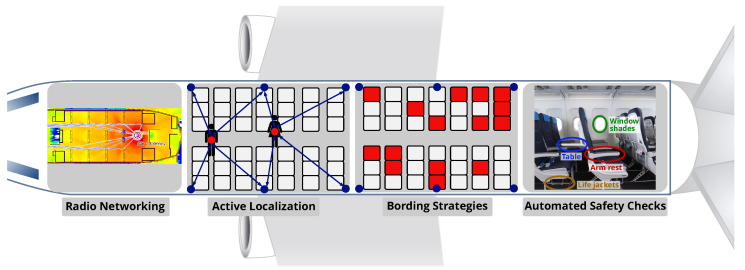
Potentials of integrated communication, localization and sensing for connected cabins.

Given these capabilities, the application of integrated WSNs within the connected aircraft cabin provides considerable operational potentials, such as dynamic control of passenger boarding or disembarkation sequences under pandemic requirements [14,15]. Figure 1 presents application areas outlining the joint use of communication, localization and sensing for aircraft operations. In this paper, we address two of these potentials using radio sensing: seat occupancy detection and the automation of passenger safety checks/announcements.

Enabling seat occupancy detection can be used to streamline the boarding process and improve the reliability of boarding time prediction [16]. Strategic decisions made before and during aircraft boarding could reduce the passenger boarding time and thus aircraft turnaround time and increase the reliability of the turnaround. In this context, the implementation of new technological approaches must always be accompanied by operational process adaptation so that the theoretical potential can be exploited (Side-Slip Seat [17,18]).

Another application of radio sensing in the context of the connected aircraft cabin is the automation of passenger safety announcements and checks; for example, the state detection of the table, the arm rest, the seat position, the window shades or the degree of filling of the hand carry baggage bins. Further, an automatic detection of missing/misplaced safety equipment using radio sensing can improve the efficiency of flight preparation.

### 1.1. Status Quo of Sensors inside the Connected Cabin

To facilitate passenger operations in the aircraft cabin, a network of sensors is needed to detect and monitor positions and movements. Required sensors are already used in various fields and could be adapted for the aircraft environment. New technologies should also be examined for their potential applications—e.g., signals from passenger devices could be used for localization—and this could reduce the amount of equipment needed in the cabin.

Various conventional sensors and systems are used for seat occupancy detection and monitoring. The most common method is probably a pressure sensor (Force Sensing Resistor) in each seat paired with a microcontroller [19]. Passive infrared sensors (PIR) are an alternative for object state detection [20,21]. Camera-based approaches use Deep Neural Networks for detection; however, they often face legal or privacy issues [22].

To connect the different sensors in the cabin and to realize new use cases for passenger and crew communication, different wireless connectivity solutions are investigated in the context of a connected aircraft cabin. For in-flight entertainment, a 60 GHz WLAN system [23] or a 5G network [24] are considered. For the monitoring and controlling of the cabin, the reliability of the communication systems is a major challenge, enabling the application of an ultra-wideband communication system [3].

In terms of radio signals, active localization based on the Received Signal Strength Indicator (RSSI) or other signal properties such as time of arrival or angle of arrival within a dense sensor network is used to locate an object or person wearing a tag [25,26]. Here, the estimated ranges between the mobile tag at the object and the fixed anchors in the WSN feed different localization methods and algorithms to ensure high accuracy and robustness. Another option is the deployment of a radar system with dedicated hardware in the cabin to passively detect the target; for example, with a mmWave 60 GHz radar [27] or an FMCW radar [28].

Overall, the aircraft cabin comprises a complex and challenging environment for radio systems due to its shape, confined space and metallic materials [2,3,29]. This leads to adverse propagation phenomena, which degrade the performance of the connection quality and localization accuracy. However, this also holds potential for multipath-enabled radio sensing and therefore enhances context and location-aware services.

### 1.2. Radio Sensing for Connected Cabin

Although there are plenty of research works focusing on radio sensing for different applications [9,10,12,13], radio sensing has not yet been discussed for the aircraft cabin. Compared to the aforementioned seat-individual sensors for detecting seat occupancy states or other safety-relevant elements in the cabin, radio sensing has several significant advantages. Following the integrated design of different purposes and features with a common signal design, no dedicated hardware is required for the detection. The usage of different signal properties allows an estimation of the propagation inside the wireless communication channel. While being the most dominant challenge for active localization systems [30,31], multipath and non-line-of-sight propagation actually enable the application of the multipath-assisted radio sensing (MARS) system [32,33,34].

By accessing the necessary information provided by the radio modules, multi-purpose, spectrum and hardware-efficient surveillance and state monitoring can be enabled while still maintaining the communication capabilities. In addition, this also increases the energy efficiency, while lowering the general infrastructure and associated costs for dedicated sensors.

The key component behind the work presented is built on the usage of the channel impulse response (CIR). Given transmitted, time-discrete impulses, the received CIR describes the communication channel in the time domain as it consists of the superposition of signal paths. MARS is based on the assumption that lengths of individual reflection paths are represented in the CIR and can therefore be geometrically mapped to the environment, enabling passive object sensing [32,34].

**Figure 2:**
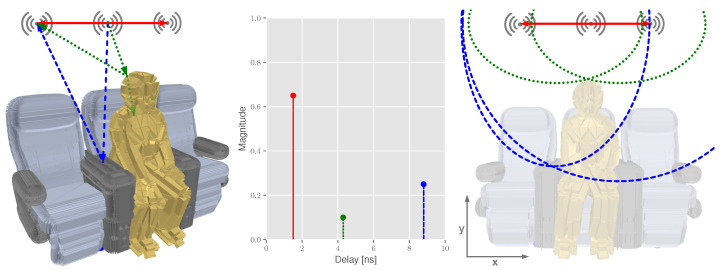
Multipath propagation in the aircraft cabin: 3D model of a seat row including a passenger, the theoretic CIR and the geometric interpretation using ellipses. Direct paths are given in red, reflection paths in green and blue.

This key idea within the context of the discussed application is briefly given in Figure 2. Here, radio modules are located above each individual seat, possibly serving a dedicated high-speed data link. Assuming a symmetrical constellation, the measured direct path distance from the central device to both outer devices is represented in red. Naturally, the shortest received signal path in the CIR represents the direct path. In addition, reflections caused by the interior (blue) or passengers (green) are also measured. As metallic or plastic materials provide better reflection properties, reflections caused by those objects might have a longer reflection path but are received with less attenuation when compared to a human body reflection. These reflections are mapped to the profile of the scene and geometrically represent individual ellipses.

Due to its enhancements in context and location-awareness, radio sensing is also a key driver of innovation for next-generation radio networks, enabling a wide range of new use cases. In particular, joint communication and sensing systems [13] for Beyond 5G (B5G) systems [10] are planned to open up new opportunities. Fifth-generation systems already use the mmWave spectrum with a bandwidth of up to 400 MHz, and 6G is expected to operate in the terahertz/submillimeter spectrum with even higher bandwidths and will therefore enable high resolutions for radio sensing.

### 1.3. Focus and Structure of the Article

This article focuses on the implementation of the MARS approach for state and occupancy detection in the connected aircraft cabin and enhances already established state detection methods with respect to the challenges of the cabin environment. The basic framework for deriving spatial information from measured CIR was presented in [32], whereas we also previously applied radio-based occupancy detection for smart parking applications in [35]. In contrast to these works, and in order to address the challenges in the complex aircraft environment, we apply both non-parametric stochastic filtering and k-nearest neighbor classification (kNN) for state detection. The latter is commonly applied to familiar localization problems, such as RSSI Wi-Fi [36] or Bluetooth [37] fingerprinting, as well as CIR data [34,38]. In contrast to these and previously mentioned works, we identify the following major contributions of this work:

- The discussion of the applicability of radio sensing within the connected aircraft cabin, including passenger seat occupancy detection for boarding monitoring and automated cabin and passenger safety checks.
- The derivation and application of CIR-based state detection methods for this application, including probabilistic filtering for spatial mapping and kNN.
- An empirical measurement survey using Ultra-Wideband devices using an aircraft seat mockup in both an anechoic chamber under close to ideal conditions and a laboratory environment in order to evaluate the proposed methods.

The remainder of the article is structured as follows: after the introduction (Section 1) and a brief problem formulation in Section 2, we discuss the MARS approach detailing methods for mapping the CIRs, the detection of objects and a CIR classification in Section 3. In Section 4, the underlying measurement setup is briefly introduced, followed by the results of the proposed mapping, detection and classification algorithms in different use cases. The paper concludes with a summary and proposals for future research work in Section 5.

## 2. Problem Formulation

For cooperative localization approaches in a WSN, target objects are typically equipped with an active transponder. By interpreting various signal properties, such as RSSI, time of flight or angle of arrival [39], geometrical relations can be derived. In contrast, radio sensing characteristics are not directly measured at the target object; instead, only secondary measures can be observed in particular reflections. Therefore, the underlying geometrical model differs from active radio localization and is briefly introduced.

In general, a sensor network consists of multiple transceivers. In our specific case (Figure 2), three transceivers are deployed, from which one is configured as transmitter at position at $X^{\mathrm{Tx}}$ and the others are configured as receivers at positions $X_{m}^{\mathrm{Rx}}$ (m=1,2). Since the transmitter and receiver locations differ from each over, the arrangement corresponds to a bistatic setup [40]. In combination with the lengths of the direct Line-of-Sight (LOS) path $d_{m}^{\mathrm{LOS}}$, the lengths of the reflection paths $d_{m}^{r}$ are calculated with the help of the reflection point X at the target. The individual positions are given in Equation (1). Please note that the given local coordinate systems refers to the profile of the aircraft seat group as given in Figure 2. The calculation of the direct paths is given in Equation (2), and the reflection paths are given in Equation (3). Geometrically, Equation (3) provides a family of ellipses, where the individual transmitter and receiver pairs are located in the focal points [32] (Figure 2).

(1) $$X^{\mathrm{Tx}}\;=\;{\left(x^{\mathrm{Tx}},y^{\mathrm{Tx}}\right)}^{⊺}\;X_{m}^{\mathrm{Rx}}\;=\;{\left(x_{m}^{\mathrm{Rx}},y_{m}^{\mathrm{Rx}}\right)}^{⊺}\;X\;=\;{\left(x,y\right)}^{⊺}$$

(2) $$\begin{array}{cc} \forall m:\;d_{m}^{\mathrm{LOS}} & ={∥X^{\mathrm{Tx}}-X_{m}^{\mathrm{Rx}}∥}_{2} \end{array}$$

(3) $$\begin{array}{cc} \forall m:\;d_{m}^{r} & ={∥X^{\mathrm{Tx}}-X∥}_{2}+{∥X_{m}^{\mathrm{Rx}}-X∥}_{2} \end{array}$$

In the time domain, these geometrical interrelationships can be observed by interpreting the travel time of transmitted signals using the speed of light *c*. Whereas the majority of time of flight-based ranging and localization modules only provide the estimated direct path between radio modules, the usage of simulations or more sophisticated hardware can also provide the CIR. The CIR h(t) consists of *J* time-shifted and attenuated received impulses $\delta (t\;-\;\tau_{j})$, where each δ(·) represents a possible multipath component [41]:

(4) $$h\left(t\right)=\sum_{j=1}^{J}\alpha_{j}\delta \left(t-\tau_{j}\right),$$

where $\alpha_{j}$ and $\tau_{j}$ denote the impulse amplitude and reception time delay. The transmitted signal is affected by various effects of the communication channel and the transceivers, including noise, path loss and multipath caused by reflections and scattering. The characteristics and behavior of the channel are described by CIR. In particular, the multipath propagation of the signal and the corresponding time delays and path losses reveal reflections within the propagation environment.

Most commonly, the underlying CIR is directly measured in time domain. However, available bandwidth is crucial. This is due to the inversely corresponding time resolution of the CIR. Simply speaking, the time resolution determines the interval of available samples in the CIR and therefore the ability to recognize and distinguish between individual reflection paths. Thus, wideband systems are generally better suited for sensing applications [13].

**Figure 3:**
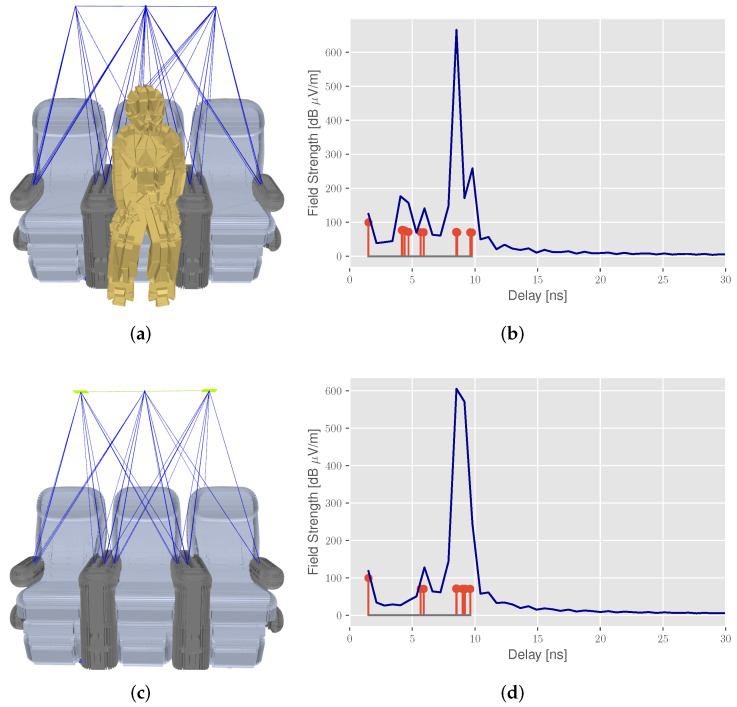
Radio propagation simulation with deterministic ray tracing to explore the multipath propagation of signals in the aircraft cabin: (**a**) seat 2 occupied with (**b**) corresponding CIR, (**c**) reference measurement (no seat occupied) with (**d**) corresponding CIR. The CIR plots show the propagation paths or multipath components (red) and the bandlimited reconstructed CIR (blue line).

In order to assess the underlying propagation phenomena, we briefly introduce the usage of radio propagation simulation [42], which we have already applied for the connected aircraft cabin in previous studies [2,29]. The scenario-based simulation offers the opportunity to obtain a better understanding of the signal propagation and reflections in the environment. In addition, this allows the optimization of parameters such as the number and position of the sensors or the influence of different objects and materials. Figure 3 presents the output of a deterministic ray tracing simulation, including the computation of spatial propagation paths (blue in Figure 3b,c) within the environment and their corresponding time delays in the CIR (red stems in Figure 3b,d).

Furthermore, we provide a bandlimited reconstructed CIR (blue line) based on a previously presented work [43], using a sinc-interpolation based on the Whittaker–Shannon interpolation formula. In the first scenario (occupied seat), the CIR in Figure 3b reveals additional reflections with a delay of about 5 ns, caused by the passenger model. These reflections from the target have a higher path loss, due to the higher transmission attenuation of the person’s skin, resulting in smaller peaks in the CIR. Without seat occupancy in Figure 3c, only seat reflections with a delay of about 8 ns are observed. In addition, the challenges in path distinction are highlighted when comparing the unlimited and bandwidth-limited CIR, as propagation paths with similar time delays are merged due to the limited time resolution of the reconstructed CIR.

## 3. Multipath-Assisted Radio Sensing (MARS)

**Figure 4:**
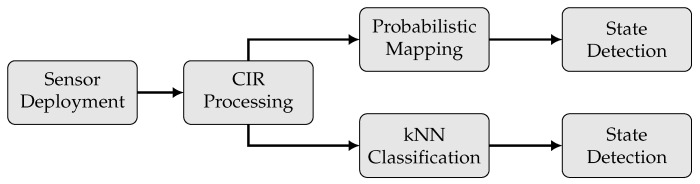
Flowchart of data processing and computation steps for radio sensing state detection.

In order to assess the surveyed CIRs as an input for radio sensing state detection, additional deployment, processing and computation steps are required, which are briefly introduced in this section and discussed later on. Given a set of sophisticated radio transceivers, which are presented in Section 4.1, the raw CIR measurements are the essential input for the discussed MARS method (Figure 4).

### 3.1. Channel Impulse Response

**Figure 5:**
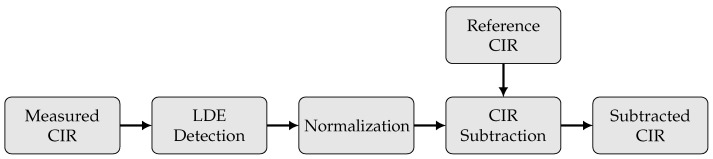
Detailed computation steps of CIR processing.

In order to process the surveyed input, additional preprocessing steps are performed to prepare the CIR for mapping and classification methods. These steps are depicted in Figure 5.

First, the time index of the CIR is aligned at the direct LOS path (Equation (2)) between the transmitter and receiver. For this task, a leading edge (LDE) detection using a dynamic threshold value is applied [34,44] with the aim of synchronizing the CIR at the direct path. In a second step, the CIR magnitude values are normalized to ensure a better comparability between the different CIR measurements. The normalization of the CIR data h(t) is done via the min–max method [45]:

(5) $$\bar{h}\left(t\right)=\frac{h(t)-\min h(t)}{\max h(t)-\min h(t)}.$$

Consecutively, a background subtraction is applied to distinguish between reflections at the target object and static background reflections. Conventionally, this method is commonly applied for image analysis to isolate moving objects in a video [46]; however, it was applied to radio sensing in our previous work [35]. The key idea is to provide a reference CIR $h^{\mathrm{ref}}\left(t\right)$ representing an empty scene with only static objects (Figure 3c,d). This reference CIR is then subtracted from the current measured CIR ${\bar{h}}_{t_{s}}\left(t\right)$ at the measurement epoch $t_{s}$:

(6) $${\bar{h}}_{t_{s}}^{\mathrm{sub}}\left(t\right)=\left|{\bar{h}}_{t_{s}}\left(t\right)-h^{\mathrm{ref}}\left(t\right)\right|.$$

**Figure 6:**
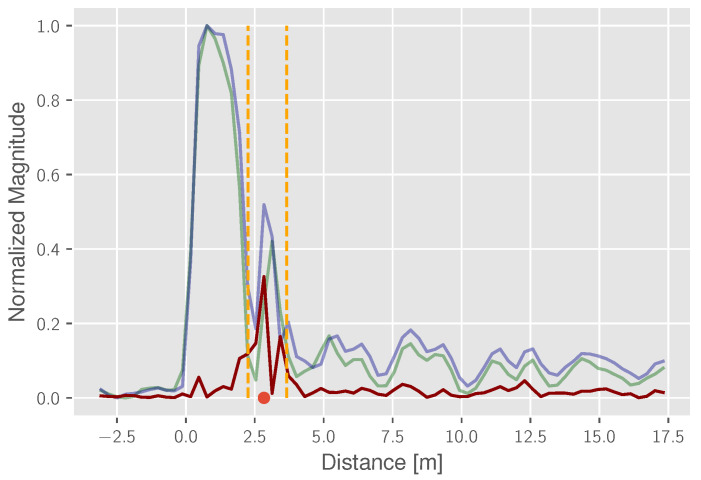
Subtraction and reflection path estimation: CIR with the object (blue), static background CIR (green), subtracted CIR (red), detection corridor (orange) and estimated reflection path (red dot).

Due to the complexity and spatial ambiguity of reflection sources in real-world scenarios, radio sensing would normally either require more bandwidth or higher infrastructure expenses. However, applying the subtraction computation, changes within the scene are easier to detect. An example for this is given in Figure 6, which was obtained from the conducted measurement campaign. It provides the comparison between the $h^{\mathrm{ref}}$ (green) and an exemplary scenario with a detectable target object (blue). The reference reflection path length at the target object is also highlighted (red dot) and is calculated by the given arrangement of the sensors using a maximum estimator. Based on this reference, a time interval (orange) is indicated, where the location and the width of the interval is dependent on the constellation as well as the size and shape of the reflecting object. Finally, the resulting, subtracted CIR ${\bar{h}}_{t_{s}}^{\mathrm{sub}}\left(t\right)$ is given in red.

Comparing the reference CIR and the surveyed CIR, a reflection distinction is not achievable. However, the proposed subtraction reveals two characteristic peaks within the expected time interval, indicating the presence of a non-static reflection object.

### 3.2. Probabilistic Grid Mapping

Following our previously published works in the field of radio sensing [32,35,47], the initial goal of this work is to map occurring reflections to the propagation environment. In the aforementioned works, we mainly focused on applying a heatmap-based environmental mapping approach to generate an imaging-like representation. For this, averaging of multiple CIR epochs is applied. With the detection task at hand, several challenges for this method arise:

- The determinacy of the equation system: with only two transmitter–receiver relations for each seat row and the assumption of a two-dimensional unknown target position X, the ellipses intersection is ambiguous.
- Multi-modality: due to the aforementioned ambiguity, in combination with a poor geometric constellation, multi-modalities in the state space are likely to occur, which hurt the presumptions of parametric state estimation.

**Figure 7:**
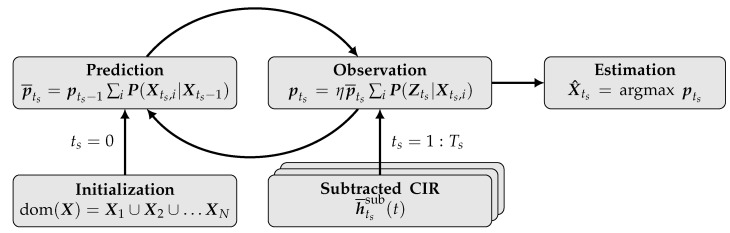
Flowgraph of probability grid mapping.

Therefore, we suggest applying a non-parametric, grid-based state estimation approach. In unison with the well-studied Extended Kalman Filter (EKF) and the Particle Filter (PF) [48], this multidimensional Histogram Filter (HF) follows the structure of the Recursive Bayes’ Filter (RBF), using a sorted sample state space representation [49]. This approach is also known as Markov Localization [50]. It follows a recursive structure consisting of a prediction and correction step based on the Markov assumption (Figure 7), incrementally estimating the hidden state space vector $X_{t_{s}}$, which represents the two-dimensional position of the reflecting object at timestep $t_{s}$, based on the last given state $X_{t_{s}-1}$.

The basic concept of this filter is to apply a discrete state space, decomposing the state space into a finite amount (*N*) of samples, also referred to as bins or grid $x_{i}$ [49]. Given this discrete representation, the prediction and correction structure of the RBF can be applied as given in Figure 7. This notation includes the predicted ${\bar{p}}_{t_{s}}$ and the resulting $p_{t_{s}}$ likelihood, based on the observation likelihood function $P(Z_{t_{s}}|X_{t_{s},i})$ and the normalization constant η. The calculation of the observation Likelihood is detailed in Algorithm 1.
**Algorithm 1:** Probability Grid Mapping—Likelihood Calculation.
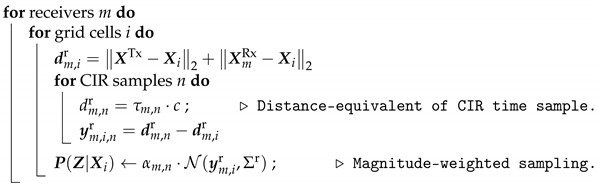


The key idea is to map each individual sample *n* in the subtracted CIR based on the normalized magnitude of the value using the elliptical representative. This is achieved by computing the length of the theoretic reflection paths for each transmitter–receiver pair and each bin and comparing these with each CIR sample. The resulting residuals y are then weighted with respect to the magnitude α sampled from a probability density function. In Algorithm 1, this is done by sampling from a normal distribution $N(\;\cdot \;,\Sigma^{r})$, where $\Sigma^{r}$ is empirically set. Finally, the estimation of the position ${\hat{X}}_{t_{s}}$ is based on the probability grid, where the bin with the highest likelihood is selected (maximum a-posteriori).

**Figure 8:**
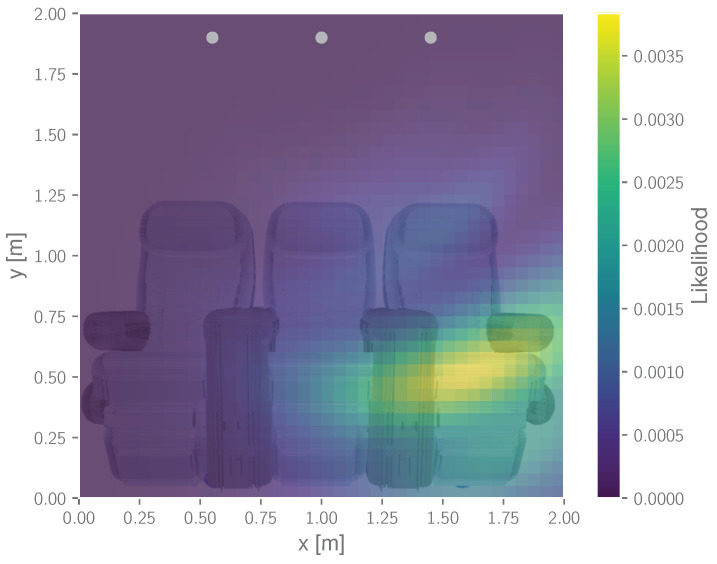
Exemplary probability mapping output for a seat occupancy detection scenario.

Similar to the previously discussed heatmap of the environment [32], this grid mapping allows us to spatially represent reflection areas and therefore indicates the location of reflecting objects. However, unlike the heatmap method, it does not require any assumptions on the underlying probability density function [49] and therefore provides a more sophisticated state estimation in the presence of multi-modalities. Figure 8 depicts an exemplary output of the mapping approach given the previously introduced cabin example.

### 3.3. kNN Classification

In addition to the previously introduced mapping method, a classification of the CIR time series is also discussed. By applying supervised learning based on predefined and labeled classes, the entire channel information stored in the CIRs can also be used for state detection.

**Figure 9:**
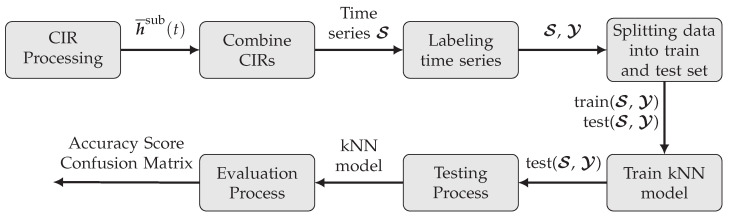
CIR kNN classification flowgraph.

For this classification task, a kNN method is implemented. The classification is computed from a majority vote of the k-nearest neighbors for each measured CIR following a defined distance metric. The model is trained by saving examples for each scene from a labeled training dataset. This approach is similar to previously investigated kNN applications—e.g., [34], where measured CIRs were assigned to a location based on fingerprinting. However, in this work, the kNN classification is not used to directly perform a localization but rather to classify the observed state. The derived kNN algorithm and its processing steps to obtain the detection state of the scene are outlined in Figure 9.

The input is the normalized (Equation (5)) and subtracted (Equation (6)) CIRs over multiple classes with respect to different use cases and scenes. For this, the CIRs of multiple receivers are combined to a single time series S. This time series is then labeled with the corresponding detection state Y for training and evaluation.

**Figure 10:**
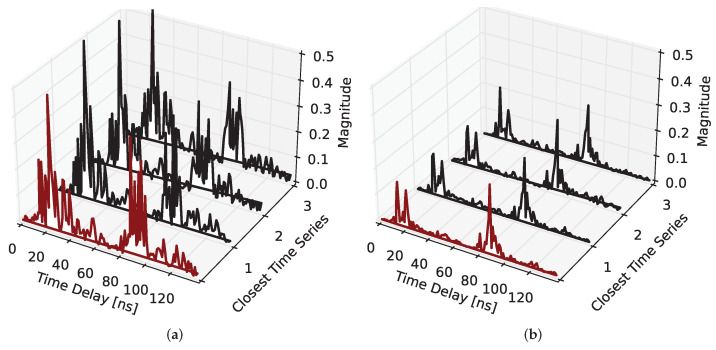
Example time series of the testing dataset (red) with the three closest neighbors from the training dataset (black) considered for the classification: (**a**) seat 1 occupied by a passenger (IV) and (**b**) Table 3 in use (I).

To fit the classifier to the training data, the measured time series of each dataset are split into a test and training data subset with a defined size. The number *k* of nearest neighbors is configurable as a parameter of the classifier and controls the number of nearest neighbors used for assigning the class in the testing stage. Figure 10 shows two examples of a combined CIR time series from the test dataset (red) as well as the four nearest neighbors from the training dataset.

Generally, the kNN classification performance depends on the following parameters, which are further discussed in Section 4.3 with respect to the surveyed datasets:

- The quality of the input data and noise in the training dataset;
- The number of classes in the dataset;
- The size of the training dataset;
- The number *k* of nearest neighbors considered for the classification;
- The choice of the distance metric.

The kNN was implemented using the *KNeighborsClassifier* provided by the Python Machine Learning package *scikit-learn* [51].

## 4. Results and Discussion

### 4.1. Measurement Setup and Datasets

**Figure 11:**
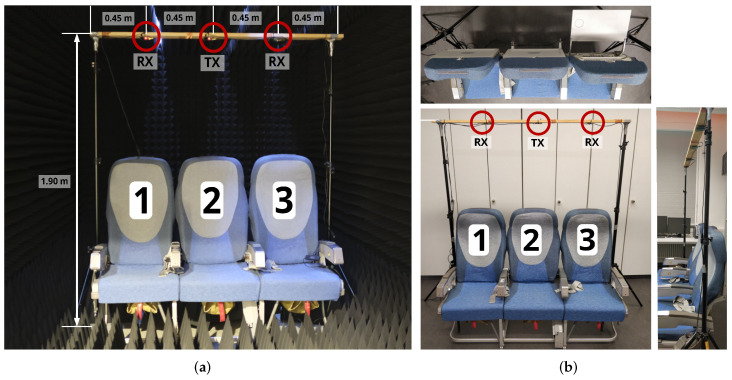
Measurement setup with one transmitter and two receivers: (**a**) in the RF anechoic chamber and (**b**) in the lab mockup. Applied seat/table numbering is also indicated.

Measurements are carried out in a radio frequency anechoic chamber (Figure 11a) under near-ideal conditions, as well as in an aircraft seat mockup in a laboratory environment (Figure 11b). Due to its inherent properties, the anechoic chamber eliminates propagation effects caused by the environment; e.g., by walls, the floor or the ceiling. Instead, only the desired target objects influence the signal propagation and generate multipath components. In addition, a more complex scenario is provided in the lab mockup, as additional reflection sources are not blocked out.

The radio modules applied in this paper are the *Decawave* DW1000 Ultra-Wideband (UWB) IC on the TREK1000 board [44,52]. Conventionally, these modules are intended to be used for ranging and localization applications; however, CIR measurements are also available, possibly enabling the discussed MARS approach. Furthermore, UWB features a bandwidth of B=500MHz, which is comparable to future radio networks (B5G), where radio sensing is a key feature. These bandwidths are beneficial for sensing applications; however, deployable B5G hardware is not yet available. The underlying property is the time resolution of the CIR, which can be expressed as $R_{time}=\frac{1}{2B}$ and therefore depends on the signal bandwidth *B*. A higher range resolution allows a better recognizability and distinction between different multipath components in the CIR, essentially improving both CIR mapping and classification.

Figure 11 shows the arrangement of the radio modules in the two chosen measurement environments. The seats and tables are numbered from 1 on the left side to 3 on the right side. For every class or scene, 200 CIRs at both receivers are measured in a near-static measurement environment.

The measurement campaign features different use cases, environments and detection scenes. The measurements were taken sequentially. An overview of the surveyed datasets, numbered with Roman numerals throughout the paper, is given in Table 1.

### 4.2. Probabilistic Grid Mapping

**Figure 12:**
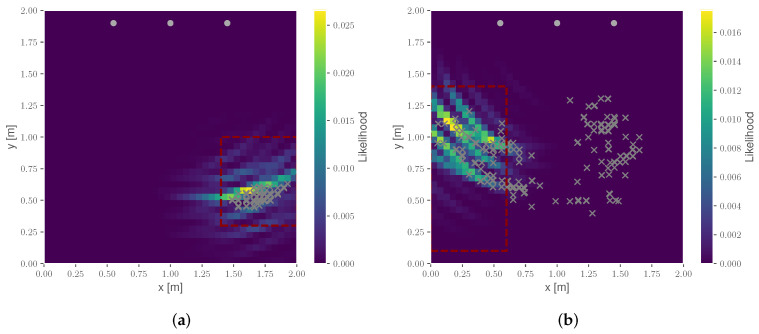
Grid-based mapping and detection: (**a**) mapping with Table 3 in use (dataset I) and (**b**) mapping with seat 1 occupied (dataset IV). Estimated positions for all observation steps (grey crosses), sensor position (grey dots) and detection area (red). In addition, the probability map is displayed, including the Likelihood result of the last observation step.

The probabilistic mapping approach is evaluated based on the detection rate of the scene. For this, a threshold detection area of both use cases and three seats is defined. An example is marked with the red dotted rectangle in Figure 12. Based on the state estimation shown in Figure 7, a boolean detection is performed, checking whether the location of the current state estimate is located within the bounds of the threshold. The detection rate then specifies the number of estimated positions over all observation inside this detection area in relation to the total number of measurements.

In the following, two scenarios are briefly discussed. At first, the detection of Table 3 in use is presented in Figure 12a. Here, all estimations are located within the detection area, and therefore an empirical detection of the table is given. In contrast, the result of the detection of a person occupying seat 1 is depicted in Figure 12b. Here, an ambiguous estimation result is presented, which over the observation time converges towards the actual occupation area. This is achieved by the recursive filtering; however, it results in an overall lower detection rate. The overall results of the mapping detection are given in Table 2.

The key advantage of the probabilistic grid mapping is the capability to potentially map reflections to the spatial domain in order to understand the origin of different peaks in the CIR. For the surveyed datasets, the detection of the seat occupancy generally has a higher success rate compared to the detection of the table scenes. However, Table 2 also unveils an inconsistent detection and a strong variation between the different detection scenes.

These observations potentially have several causes, starting with the density of the deployed sensor network in the measurement setup. Due to the aforementioned missing redundancy in available measurements, as well as the poor geometric constellation, reliable positioning is not achievable. However, the mapping would greatly benefit from more sensors to resolve ambiguities and multiple detected reflections in the CIR. Another potential source of error is the LDE detection in the CIR. If the estimated impulse reception time of the direct LOS path is incorrect, the following reflection delays cannot be properly mapped.

In conclusion, the robustness of the state detection using the mapping approach in the given seat row selective scenario is not sufficient to provide a reliable state detection in the given application context.

### 4.3. Classification

To overcome the shortcomings of the mapping method in the position domain, the performance of the kNN classification in the measurement domain is further discussed. For this task, different evaluation parameters are considered to analyze the performance of the kNN classification. This includes the accuracy score of the testing dataset combined with the confusion matrix, indicating the error rate for different detection classes/scenes, as well as kNN parameters, such as the applied distance metric and size of the training dataset.

**Figure 13:**
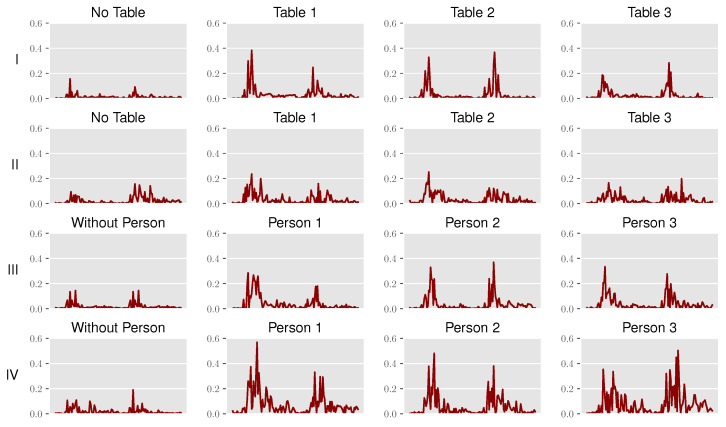
Example of CIR time series for each dataset (I–IV) across the different detection scenes.

Figure 13 depicts the input data for the classification, pointing out the specialties for every dataset and detection scene. The input is the obtained time series over multiple measurement iterations, following the discussed preprocessing steps for both receivers.

As the reference CIR is obtained from an averaged set of CIRs, differences after subtraction can still be observed even in empty scenes, and this therefore provides a good understanding of the prevailing noise floor.

In addition, the peaks in the CIRs indicate characteristic reflections for the scenes with a table in use or an occupied seat. Following the expectations, stronger peaks are observed on the side where the target objects are present. If the detection is to be expected in the middle, the measured reflections are about the same for both receivers. Despite the subtraction, the noise floor and number of reflections in the lab mockup are much higher compared to the anechoic chamber. The additional reflections at the walls, ceiling and floor can therefore not be filtered completely out by the static background subtraction.

**Figure 14:**
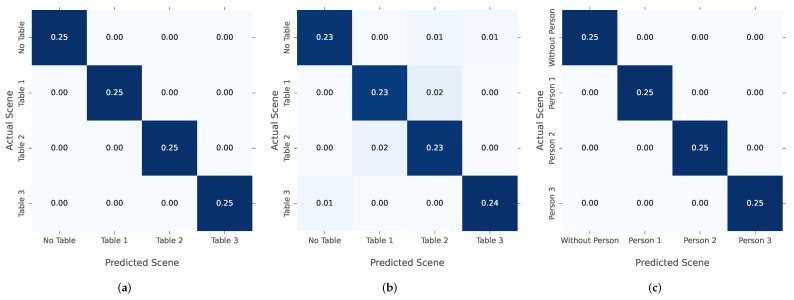
Confusion matrix for the classification: (**a**) table detection in the anechoic chamber (I a); (**b**) table detection in the lab mockup (II a); (**c**) seat occupancy detection in the anechoic chamber (III).

In order to further evaluate the performance of the kNN classification, the confusion matrix of each use case and environment is computed. For this, the confusion matrix of datasets I, II and III in Figure 14 compares the actual detected scene with the kNN-predicted scenes. The values correspond to the relative number of actual or predicted classes in relation to the size of the testing dataset. For datasets I, III and IV, a 100% prediction accuracy has been achieved. Solely in II, some scenes are predicted incorrectly. This evaluation is based on this configuration: a test set size of 50% for each dataset, k=3 and the Euclidean metric. This is discussed further later in this work.

Table 3 evaluates the accuracy score of all datasets. The accuracy is specified separately for the train and test dataset in order to recognize overfitting or underfitting of the model.

To further challenge the kNN algorithm, the number of features for some datasets is extended. For this reason, the table detection dataset is extended with scenes, where multiple tables are in use. Datasets I b and II b are the extended versions of the table detection in the anechoic chamber to six classes, or in the lab mockup to eight scenes. These additional classes also cover the detection of multiple tables in use at the same time. The results in Table 3 reveal a correct classification for all scenes in dataset I b and no increase in false detection in dataset II b compared to dataset II.

With respect to the aforementioned kNN evaluation parameters, the quality of the input data is highly dependent on the measurement environment, the sensor and use case. Therefore, the different parameters of the kNN algorithm are examined in more detail.

Generally, the background noise is lower in the anechoic chamber compared to the lab mockup, which is also supported by the measured time series in Figure 13.

**Figure 15:**
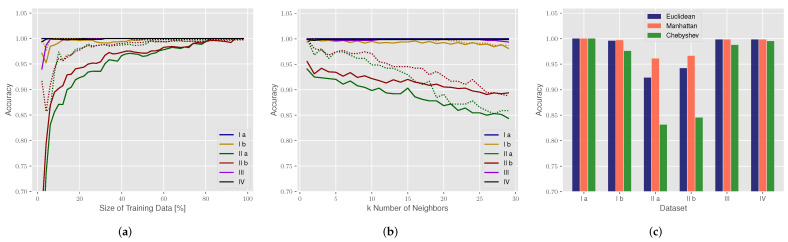
Influence of different parameters on the accuracy of the kNN classification: (**a**) size of the training dataset relative to overall dataset size; (**b**) number *k* of neighbors; (**c**) distance metrics. Accuracy of the testing data (solid line) and accuracy of the training data (dotted line).

By default, the training size is set to 0.5, k=3, and the Euclidean distance is used. The correlation between the size of the training dataset and the accuracy is shown in Figure 15a and indicates that a higher percentage of training data will increase the accuracy.

For the number of k-nearest neighbors in Figure 15b, the accuracy of the training data (doted line) is k=1, because each sample is using itself as a reference. However, this is also an overfitting case, where new unseen data cannot accurately be classified by the kNN algorithm. For the majority of datasets, the number *k* has no influence on the accuracy; only for the table detection, in the lab mockup, the accuracy dropped with a higher number of k nearest neighbors (Figure 15b).

The last parameter is the kNN distance metric, which calculates the differences between the time series and is shown in Figure 15c. Overall, three different metrics are evaluated including the Euclidean distance, Manhattan metric and Chebyshev distance [37]. Here, the Euclidean distance and the Manhattan distance provided the highest accuracies.

The accuracy of the kNN classification for all datasets is sufficient to match the requirements of the use case of boarding monitoring or automated passenger announcements and checks. When compared to the previously discussed grid mapping, kNN classification provides superior detection results. The main reasons for this are both the discussed limitations of position domain detection and the characteristic time series profiles for each scene (Figure 13). The latter avoids the issues with geometric ambiguities and can therefore perform a more reliable state detection. In conclusion, given a characteristic propagation environment with the corresponding channel information, the provided CIRs can successfully be used to train a kNN model and result in a potent state detection performance. One drawback of kNN classification is the handling of new unseen detection scenes, as the model must first be generalized with more training data to avoid overfitting.

## 5. Conclusions and Outlook

In this contribution, an integrated communication and sensing system for different use cases in the context of a connected aircraft cabin was presented. The use cases included seat occupancy detection for boarding monitoring and general state detection as a sensory input for the automation of passenger announcement and checks. In particular, the MARS approach utilizes multipath information of the signal in the CIR.

In this paper, an approach that processes the CIRs in order to identify the given states was presented. This included several preprocessing steps, such as LDE detection, normalization and static background subtraction, providing the differences between the surveyed and a reference CIR. Given this preprocessing, two state detection approaches were presented: a probabilistic mapping approach based on the RBF and a kNN approach. Given the introduced application field and its corresponding boundary conditions, the kNN approach provided reliable classification rates and therefore enabled a robust state detection based on an empirical measurement campaign in both an anechoic chamber and a laboratory mockup. Our results demonstrate the suitability of a radio sensing system for the new application area of a connected aircraft cabin but should also be extended to a broader range of measurement scenes in a real aircraft environment.

**Figure 16:**
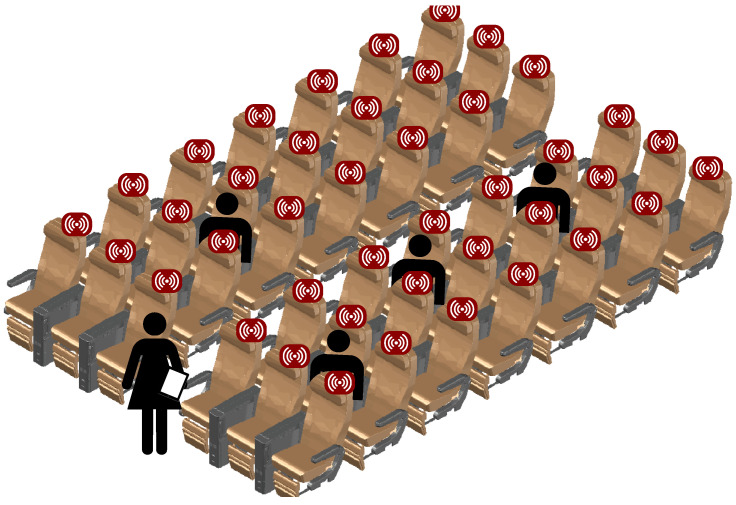
Future connected aircraft cabin system for communication, localization and sensing.

In the future, the discussed MARS system for the connected cabin can greatly benefit from large signal bandwidths and an increased density of sensors to enable a small cell configuration. Therefore, Figure 16 depicts a schematic outlook on these integrated systems, scaled up to an entire aircraft cabin. Ultimately, the synergy of future, digitized customer services (e.g., personalized, high data rate passenger entertainment) and its double-use as a sensory basis for operational management and aircraft maintenance will be the main selling point for integrated communication and sensing systems in the connected cabin.

However, this environment is still very demanding and therefore requires further investigations. Given the complexity of the scenarios and the occurring propagation effects, ambiguities can potentially be even more critical in a scaled system. In particular, the differentiation of multipath components from the target objects and the surroundings is essential for a successful detection state estimation. Besides new advanced algorithms [53], the adaption of electromagnetic properties of the surrounding material surfaces is one option to cope with these challenges. One focus of further research is therefore the optimization of the propagation environment for radio sensing to eliminate undesirable reflections from the surroundings by using special material structures. An example for this can be the equipping of target objects (table) with reflective metallic layers in order to generate a more characteristic reflection profile when being in use. Potentially, this approach can be reversed for the passenger detection to accelerate the attenuation cased by the person compared to an improved reflecting seat surface.

In addition, further research should focus on an extension of use cases for radio sensing in the connected aircraft cabin. For example, the detection of the filling degree of the hand luggage bins can further streamline the boarding process.

## Figures and Tables

**Table 1:** Surveyed CIR datasets for different use cases and environments.

Dataset	Use Case	Environment	Samples/Class
I	table detection	anechoic chamber	200
II		lab mockup	200
III	seat detection	anechoic chamber	200
IV		lab mockup	200

**Table 2:** Detection rates of the mapping approach for different use cases and environments.

Dataset	Table/Person 1	Table/Person 2	Table/Person 3
I	0.00	0.00	1.00
II	0.00	0.00	0.10
III	0.96	0.53	0.00
IV	0.34	0.06	0.96

**Table 3:** Accuracy scores of the kNN classification for the different datasets and use cases.

Dataset	Number of Classes	Labels/Scenes	Accuracy Train Set	Accuracy Test Set
I a	4	[No Table, Table 1, Table 2, Table 3]	1.00	1.00
I b	6	[No Table, Table 1, Table 2, Table 3, Tables 1 and 3, Tables 2 and 3]	1.00	1.00
II a	4	[No Table, Table 1, Table 2, Table 3]	0.98	0.92
II b	8	[No Table, Table 1, Table 2, Table 3, Tables 1 and 2, Tables 1 and 3, Tables 2 and 3, Table All]	0.98	0.94
III	4	[Without Person, Person 1, Person 2, Person 3]	1.00	1.00
IV	4	[Without Person, Person 1, Person 2, Person 3]	1.00	1.00

## Data Availability

Not applicable.
